# Predictive Role of Cytokine and Adipokine Panel in Hospitalized COVID-19 Patients: Evaluation of Disease Severity, Survival and Lung Sequelae

**DOI:** 10.3390/ijms241612994

**Published:** 2023-08-20

**Authors:** Laura Bergantini, Miriana d’Alessandro, Sara Gangi, Francesco Bianchi, Paolo Cameli, Beatrice Perea, Martina Meocci, Gaia Fabbri, Sofia Marrucci, Moftah Ederbali, Elena Bargagli

**Affiliations:** 1 Respiratory Disease and Lung Transplant Unit, Department of Medical Science, Surgery and Neurosciences, Siena University, 53100 Siena, Italy; laurabergantini@gmail.com (L.B.); dalessandro.miriana@gmail.com (M.d.); sara.gangi@student.unisi.it (S.G.); bargagli2@gmail.com (E.B.); 2 Pneumology Department, Azienda USL Toscana Sud-Est, “Misericordia” Hospital, 58100 Grosseto, Italy

**Keywords:** cytokine, adipokine, profiles, COVID-19

## Abstract

Coronavirus disease 2019 (COVID-19) may determine a multisystemic chronic syndrome after resolution of SARS-CoV-2 infection in a significant percentage of patients. Persistent cytokine dysregulation can contribute to long-lasting inflammation and tissue damage, resulting in the diverse, often debilitating symptoms experienced by some patients (so-called long COVID syndrome). The aim of our study was to evaluate the value of a panel of serum biomarkers of severity and prognosis in patients hospitalized for COVID-19 and also as predictive factors for the development of post-COVID lung sequelae after discharge from the hospital. All blood sampling was performed in the first 24 h after admission to the hospital. Serum analyte concentrations of IL-4, IL-2, CXCL10 (IP-10), IL-1β, TNF-α, CCL2 (MCP-1), IL-17A, IL-6, IL-10, IFN-γ, IL-12p70 and TGF-β1 were quantified by bead-based multiplex LEGENDplex™ analysis and commercially available ELISA kits. A total of 108 COVID-19 patients were enrolled in the study. Comparative analysis of these proteins showed higher levels of TGF-β and IL-6 and lower levels of RBP-4 and IL-10 in the severe group. Age, adiponectin, IL-8 and IL-32 resulted as the best predictors for survival. Moreover, IL-1β, IL17A, TNF-α, TGF-β, IL-4 and IL-6 were significantly higher in patients who showed HRCT evidence of fibrotic interstitial alterations at follow-up than patients who did not. The initial inflammatory status of patients on admission to the hospital with COVID-19, as reflected by the present panel of adipose tissue-related biomarkers and cytokines, offered insights into medium-term prognosis.

## 1. Introduction

Coronavirus disease 2019 (COVID-19) caused by severe acute respiratory syndrome coronavirus-2 (SARS-CoV-2) has posed a significant threat to global health [1,2]. Symptoms can vary from none to acute respiratory distress syndrome (ARDS) (about 15% of cases) [3]. The latter is not induced by viral load but rather by an excessive immune response, involving an exaggerated amount of pro-inflammatory cytokines and leading to what is termed a “cytokine storm” [4,5,6]. In the context of COVID-19, cytokine storms have been observed in hospitalized patients with severe disease [7]. Uncontrolled release of these molecules can lead to widespread inflammation, tissue damage and organ failure [8].

There is much information in the literature on cytokine storms and COVID-19 in hospitalized patients. However, it was recently found that in some individuals, the immune system continues to show abnormal activity even after the initial viral infection has cleared [9]. Indeed, persistent cytokine dysregulation can contribute to long-lasting inflammation and tissue damage, resulting in the diverse, often debilitating symptoms experienced by some patients [9,10,11,12]. The World Health Organization (WHO) named this specific syndrome as “long COVID” or “post-COVID-19 condition”, defining it as “the continuation or development of new symptoms 3 months after the initial SARS-CoV-2 infection, with these symptoms lasting for at least 2 months with no other explanation” [13].

Interestingly, different studies have identified certain cytokines that may be predictive of the development or persistence of long COVID symptoms. In particular, IL-6 and IL-8 have been described in concomitance with severe COVID-19. Higher IL-6 and TNF-α levels have been correlated with prolonged symptoms, while IL-8 has been associated with patients experiencing respiratory symptoms and persistent inflammation during follow-up [14,15]. IL-10, a cytokine with immunoregulatory and anti-inflammatory properties, has been reported to be low in patients with long COVID symptoms, suggesting that an imbalance between pro-inflammatory and anti-inflammatory cytokines may contribute to the persistence of symptoms [16]. However, research in this field is still evolving and more studies are needed to establish definite associations [17].

Besides cytokines, adipokines also play a crucial role in regulating various physiological processes, including metabolism, inflammation and immune function [18,19]. Emerging evidence suggests that adipokines have implications in the context of COVID-19. Adiponectin (APN) and retinol-binding protein 4 (RBP4) are considered to be anti-inflammatory molecules. Lower levels of adiponectin and RBP4 have been associated with higher risk of severe COVID-19 and adverse outcomes [20,21]. On the other hand, resistin is an adipokine involved in insulin resistance and inflammation. Higher levels of resistin may contribute to chronic low-grade inflammation. Some studies have reported elevated resistin levels in COVID-19 patients, particularly in those with severe disease [22]. However, to our knowledge, no data are available concerning the implications and the potential predictive value of adipokines for post-COVID respiratory alterations, such as the impairment of lung volumes or diffusion capacity and/or the evidence of fibrosis detected in chest imaging.

Since the role of cytokines in the acute phase of the disease is well established [23], the aim of our study was to evaluate the value of a panel of serum biomarkers of severity and prognosis in patients hospitalized for COVID-19 and also as predictive factors for the development of post-COVID lung sequelae after discharge from hospital.

## 2. Results

### 2.1. Patient Features

Demographic data, clinical data and immunological findings are reported in Table 1. There was a prevalence of males in the two groups of patients: 64% in the group with mild–moderate disease and 57% in the severe group. All patients underwent a chest X-ray in the first two days of hospitalization: bilateral diffuse pneumonia was detected in 71% (10/14) of severe patients and 61% (58/94) of mild–moderate patients (*p* = 0.04). Regarding symptoms, 98% of patients showed at least two symptoms at onset, with a prevalence of fever. In the total population, 27% of mild–moderate patients and 58% of severe patients were without specific medical or surgical comorbidities, while the others showed almost one comorbidity, of which cardiomyopathy was the most common (*p* = 0.01).

Among blood parameters, severe COVID-19 patients showed significantly lower lymphocytes and higher monocytes percentage than in the mild–moderate group. CRP was significantly higher in severe patients than in the mild–moderate group (*p* = 0.03).

### 2.2. Cytokine Levels in Relation to the Severity of COVID-19

**Figure 1:**
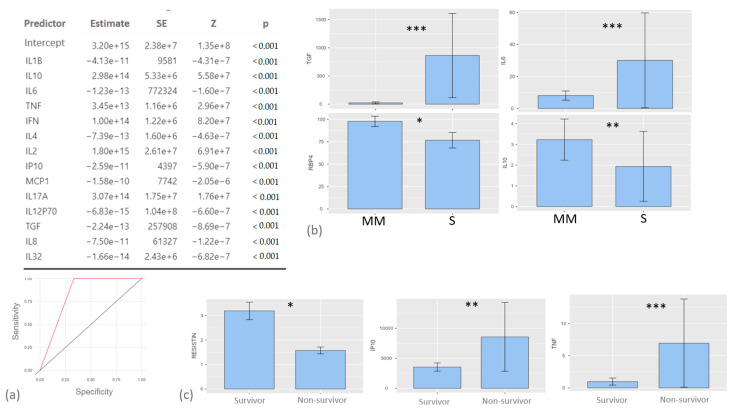
(**a**) Logistic regression model (with ROC curve) including the serum cytokines evaluated in the study with disease severity as the dependent variable. (**b**) Comparison of serum TGF-β, IL-6, RBP4 and IL-10 concentrations between patients with mild to moderate (MM) and severe (S) COVID-19 disease. (**c**) Comparison of serum resistin, IP10 and TNF-α values between survivor and non-survivor patients. *: *p* < 0.05; **: *p* < 0.001; ***: *p* < 0.0001.

The logistic regression model with severity groups as the dependent variable showed that all the analytes helped discriminate the two groups, resulting in a ROC curve with an AUC of 0.83 (*p* < 0.0001) with good sensitivity and specificity (1.0 and 0.66, respectively) (Figure 1a). Comparative analysis of these proteins showed that higher levels of TGF-β and IL-6 and lower levels of RBP-4 and IL-10 were mainly associated with the severe group (Figure 1b).

### 2.3. Resistin, IP-10 and TNF-α Concentrations Were Associated with Survival

Nine of our patients (8.3%) died in hospital. Resistin, IP-10 and TNF-α concentrations were associated with survival; in particular, higher IP-10 and TNF values were present in dead patients while resistin showed an inverse trend (Figure 1c).

**Figure 2:**
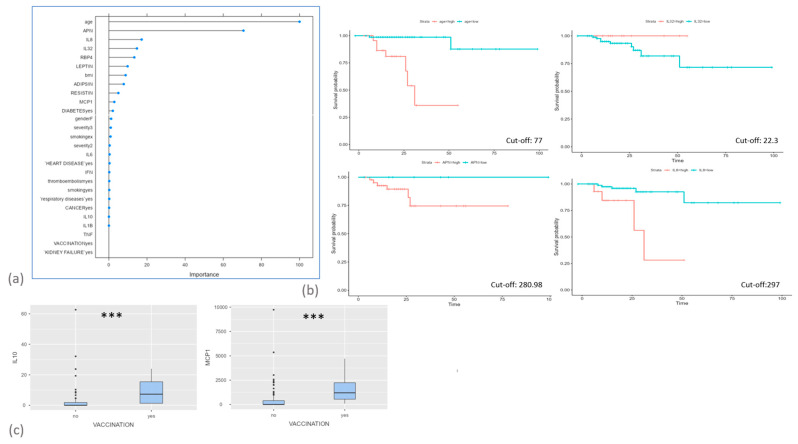
(**a**) Random forest variable importance plot for the model of survival prediction and (**b**) comparison of survival curves according to Cox regression analyses performed on the variables with the best performance (age, adiponectin, IL-8 and IL-32). (**c**) Comparison of serum IL-10 and MCP values in the study population according to vaccination status. ***: *p* < 0.0001.

After stratification of our population on the basis of survival during hospitalization, machine learning analysis with a variable importance plot was used to select the best variables for identifying the most accurate predictors. The model selected age, adiponectin, IL-8 and IL-32 as the best predictors for survival with a model accuracy of 0.85 (Figure 2a). Using this model, COX regression survival analysis was chosen to detect the best cut-off point for discriminating patients who died from those who survived. All four variables proved to be associated with the survival rate. Interestingly, increases in age, APN and IL-8 concentrations were associated with a worse prognosis, while higher concentrations of IL-32 were observed in survivors (Figure 2b). Vaccinated patients showed higher levels of MCP-1 and IL-10. No comorbidity was associated with altered levels of the cytokines analysed (Figure 2c).

### 2.4. Altered Levels of Cytokines Were Associated with the Development of Fibrosis 3–6 Months after Discharge from the Hospital

In order to establish whether some of these biomarkers were associated with HRCT evidence of sequelae after hospitalization for COVID-19, clinical and radiological data with functional parameters, including FEV1, FVC and DLCO expressed as percentages, were collected for the follow-up patients. Eleven patients, mean age 57 ± 37.4 years, prevalently males (10/11), showed HRCT evidence of fibrotic alterations, unlike the other forty-five. Of these eleven patients, nine (81%) showed air trapping and ten (90%) showed ground glass opacities. Twenty-nine of the other forty-five patients (64%) showed evidence of air trapping and thirty (66%) showed evidence of ground glass opacities.

**Figure 3:**
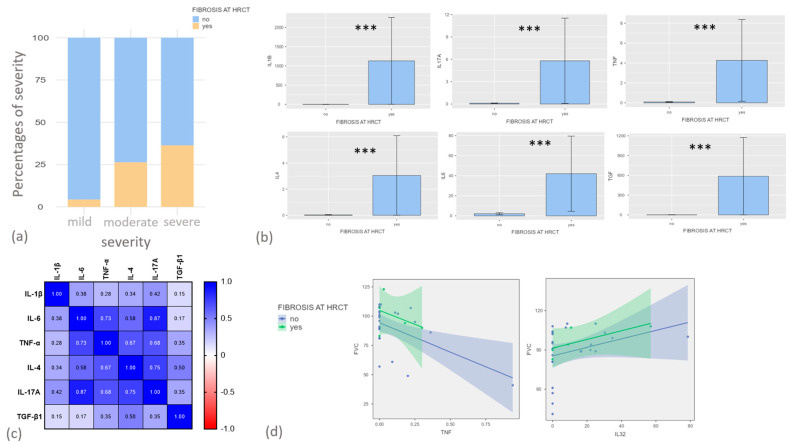
(**a**) Contingency analysis between the severity of COVID-19 and evidence of lung fibrosis during follow-up; (**b**) comparisons of serum IL-1β, IL17A, TNF-α, TGF-β, IL-4 and IL-6 concentrations between patients with or without lung fibrotic alterations at post-COVID-19 follow-up; (**c**) correlation matrix among serum biomarkers; (**d**) correlation plot between FVC (expressed as percentage of predicted value) and TNF-α and IL-32. ***: *p*< 0.0001.

As reported in Figure 3a, patients who developed severe COVID-19 during hospitalization showed a higher percentage of fibrotic sequelae in follow-up HRCT (χ^2^ = 6.06 *p* value = 0.048). Concerning serum biomarkers evaluated on admission to hospital, IL-1β, IL17A, TNF-α, TGF-β, IL-4 and IL-6 were significantly higher in patients who showed HRCT evidence of fibrotic interstitial alterations at follow-up than patients who did not (Figure 3b).

As reported in Figure 3c, these biomarkers appeared to be closely correlated with each other, showing particular significance between IL-6 and TNF-α (r = 0.73 *p* < 0.0001), IL-6 and IL-17a (r = 0.87 *p* < 0.0001) and between IL-4 and IL-17a (r = 0.75 *p* < 0.0001).

Concerning lung function parameters at follow-up, FVC% was significantly correlated with TNF-α (r = −0.42, *p* = 0.01) and IL-32 (r = 0.34, *p* = 0.01).

## 3. Discussion

COVID-19 is a disease with a heterogeneous spectrum of manifestations that vary with the age and comorbidities of the patient [24]. Several immune markers have been investigated as predictors of survival and severity of this infection, including many serum cytokines [1] but much less data are available on bioindicators with predictive values for fibrotic lung sequelae after discharge from hospital [9]. In this study, we investigated a broad panel of proteins, including cytokines and adipokines, in hospitalized COVID-19 patients to assess their predictive potential for severity of disease, survival and delayed lung complications.

In line with previous findings, logistic regression showed that all cytokines displayed good accuracy for discriminating severe patients from mild to moderate cases of COVID-19. Overall, as previously reported, our study confirmed that higher serum concentrations of pro-inflammatory cytokines and, concurrently, lower values of anti-inflammatory or immunoregulatory molecules are strictly related with COVID-19 severity and, consequently, might have a significant impact in terms of early mortality. In the present study, in examining the pleiotropic and overlapping function of serum cytokines and their mutual interrelationship, the development of a predictive model including a wide panel of analytes allows us to detect the molecule best associated with severity and mortality in our cohort. Concerning the prediction of mortality, the best performance was observed for TNF-α, IL-8 and 32 and IP-10, which were significantly higher in non-survivors; even though this was not surprising, these results highlight how the onset of an inflammatory disequilibrium is crucial for the clinical course of COVID-19 and further confirm the potential role of these cytokines as early prognostic biomarkers for hospitalized patients. The direct comparison showed that TGF-β and IL-6 were significantly higher in severe patients, while IL-10 and RBP-4 were lower. These findings are in line with the literature, which among other things reports a correlation between RBP-4 and the severity of COVID-19, stressing the clinical importance of its anti-inflammatory properties in this setting [20].

Apart from IL-10, MCP-1 was also found to be higher in COVID-19 patients who had previously been vaccinated. Our understanding of MCP-1’s specific role in the disease is evolving, although conflicting results have been reported so far. In the context of COVID-19, elevated MCP-1 levels have been associated with the recruitment and activation of monocytes and other immune cells to the lungs. These cells can contribute to the inflammatory response observed in severe cases of COVID-19 [28,29]. However in other studies, MCP-1 was described to be higher in mild COVID-19 than in severe cases, probably due to inhibition in IFN signalling [28], suggesting a protective role of MCP-1 against the onset of cytokine storms. In our population, we did not observe significant differences in MCP-1 expression between mild–moderate and severe patients: this divergence from previous reports may be due to the inclusion of a high number of vaccinated patients, which may have influenced MCP-1 values.

Interestingly, we also observed higher concentrations of another cytokine with immunoregulatory and anti-inflammatory functions; namely, IL10. This cytokine was higher in mild–moderate patients than in severe cases and also in patients who were vaccinated before contracting the disease and requiring hospitalization. Protection against tissue damage by IL-10-producing Treg cells has long been known [25]. In the acute phase of viral infections, IL-10 released from innate immune cells and effector T cells balances immune damage and defence [26]. The potential of IL-10-producing virus-specific Tregs in the treatment of human coronavirus was also recently described [27].

These results are in line with a previous paper, where we found that IL-32 was higher in healthy controls than in COVID-19 patients [23]. Interestingly, this interleukin also seemed linked to post-COVID lung function parameters, especially FVC. In fact, a significant direct correlation was observed between IL-32 and FVC percentage of predicted value.

Severe cases of COVID-19 have been associated with a higher frequency of HRCT alterations. The lungs can suffer inflammation and damage due to this viral infection [30]. In time, this can lead to the formation of scar tissue, resulting in fibrosis, which, in turn, lead to breathing difficulty and to a reduction in lung volumes and diffusion capacity [31].

Many other cytokines (released by Th1, Th2 and Th17 cells) are reported to be associated with HRCT sequelae 3–6 months after hospitalization [32]. In particular, IL-6 and IL-17A have been directly correlated with HRCT alterations, meaning that on admission to hospital, the imbalance between pro- and anti-inflammatory mediators may already not only affect the probability of survival but also the risk of developing long-term respiratory impairment. The increased levels of pro-inflammatory cytokines already detected on admission to hospital are therefore linked not only to disease severity but also to subsequent impairment of lung tissue.

Our study has many limitations: First, the monocentric design and the limited sample size surely limit our findings in terms of scientific soundness and reproducibility of data, even though, on the other hand, it ensured an at least fair standardization for the hospitalization criteria and for laboratory procedures. Second, the majority of demographic and clinical features have been collected from hospital medical records and may be prone to recalling and reporting bias. Third, different variants of SARS-CoV-2 may potentially lead to different immunological alterations and different post-COVID-19 lung sequelae in terms of frequency and severity. We decided not to stratify the study population on these terms because SARS-CoV-2 variant strains identification was not routinely performed in our centre and, therefore, we were not able to provide a reliable classification. Four, we specifically focused on post-COVID-19 lung sequelae, evaluated through radiological and functional assessment; although this choice helped to focus more specifically on respiratory issues, our findings cannot be translated to broad long COVID syndrome. Five, we did not include clinical symptoms reported by the patients at hospital admission in the statistical model for the prediction of mortality and development of lung sequelae, because the main aim was to investigate the potential of serum biomarkers on this issue and the sample size was not powered enough to include the heterogenous clinical spectrum of COVID-19. However, it surely limits the feasibility of our model, especially for those hospitals that may not have easy access to laboratory biomarkers.

## 4. Materials and Methods

### 4.1. Study Design and Participants

The study was performed with subjects 18 years of age or over who were infected with SARS-CoV-2 and hospitalized at Siena University Hospital or Grosseto Hospital between March 2020 and June 2022. All COVID-19 cases were confirmed by nasopharyngeal swabs positive for SARS-CoV-2 by reverse-transcription polymerase chain reaction (RT-PCR) and classified as mild, moderate, severe and critical according to WHO criteria (World Health Organization. Clinical Management of COVID-19. World Health Organization, 2020, pp. 1–62) [33]. Since the population was small, we pooled mild and moderate cases into a single group and severe and critical into a second group. According to centre protocol for the management of suspected COVID-19 subjects, all patients were admitted at first in the emergency room (ER) of our hospital, where they performed a nasopharyngeal swab for SARS-CoV-2 detection, lab tests and diagnostic procedures according to the clinical judgement of ER staff. To be included in the study, all blood samplings had to be performed within the first 24 h after admission to hospital. Considering the wide heterogeneity of laboratory exams prescribed in the ER setting, we decided to collect only the parameters available in the entire study population (blood cell count, including haemoglobin, haematocrit, white blood cells count with percentages of neutrophils, lymphocytes, monocytes, eosinophils and basophils, platelets and C-reactive protein (CRP). After discharge from hospital, 56 patients participated in the post-COVID-19 follow-up protocol. These cases underwent medical examination, high-resolution computed tomography (HRCT) of the chest, blood tests and lung function tests between 3 and 6 months after discharge from hospital. All data, including clinical, sociodemographic and survival, as well as follow-up lung function parameters, comorbidities, vaccinations and the main HRCT findings, including air-trapping and ground glass opacity, were entered into a database.

The study complied with the principles of the Declaration of Helsinki. The University Ethics Committee approved the study (CEAVSE PAN_HUB_2021, code number 17431_0_1). All patients gave their written informed consent to participate in the study and use their data.

### 4.2. Stratification of Patients Based on the Severity, Survival and Development of Fibrosis at HRCT during Follow-Up

A total of 108 participants were enrolled and grouped as follows for analysis purposes: mild–moderate (n = 94) patients and severe–critical patients (n = 14). Subsequently, the patients were divided between dying during hospitalization (n = 8) and patients discharged (n = 100). Moreover, the subgroup of patients with follow-up (n = 56) was stratified on the basis of HRCT evidence of fibrosis (n = 11 with and n = 45 without).

### 4.3. Analyte Detection

Serum analyte concentrations of IL-4, IL-2, CXCL10 (IP-10), IL-1β, TNF-α, CCL2 (MCP-1), IL-17A, IL-6, IL-10, IFN-γ, IL-12p70 and TGF-β1 (free active form) were quantified by bead-based multiplex LEGENDplex™ analysis (LEGENDplex™ Custom Human Assay, Biolegend, San Diego, CA, USA) according to the manufacturer’s instructions. In a subset of 62 patients, five proteins related to different pathophysiological mechanisms (adiponectin, adipsin, RBP-4, leptin and resistin) were selected for assay in the serum. Serum concentrations of the proteins were quantified in pg/mL by bead-based multiplex LEGENDplex™ analysis (LEGENDplex™ Custom Human Assay, Biolegend) according to the manufacturer’s instructions. Reactions were run in duplicate with a BD FACSLyric flow cytometer (BD-Biosciences San Jose, CA, USA). The adipokine concentrations were processed with Legendplex V8.0 software (Biolegend, San Diego, CA, USA).

### 4.4. ELISA Kit

Serum concentrations of IL-8 and IL-32 were determined using enzyme-linked immuno-sorbent assay (ELISA) kits from Invitrogen (Waltham, MA, USA) and MyBioSource (San Diego, CA, USA), following the manufacturer’s instructions. Concentrations were read at 450 nm with a Victor X4 fluorimeter (Perkin Elmer, Waltham, MA, USA) and expressed in pg/mL.

### 4.5. Statistical Analysis

To describe the study population of COVID-19 patients in terms of sociodemographic and clinical characteristics, non-parametric Mann–Whitney tests were used for continuous numerical variables. For comparison of the relative frequencies of the different levels of nominal/categorical variables, Fisher’s exact tests were used. Multivariate analysis with a machine learning approach, based on the “caret” package (short for Classification And REgression Training) was performed. The model was designed to select the variables best associated with survival, whose statistical evaluation was conducted through Cox regression analysis. Correlations were determined by using the Spearman correlation coefficient. Probability values less than 0.05 were considered statistically significant. Statistical analysis was performed with GraphPad Prism 9.2 software and Jamovi free software version 2.3.26.

## 5. Conclusions

In conclusion, the possibility of detecting patients at risk of developing lung damage by monitoring inflammatory proteins on admission to hospital would evidently help in the management of these patients. The initial inflammatory status of patients on admission to hospital with COVID-19, as reflected by the present panel of adipose tissue-related biomarkers and cytokines, proved to offer insights into medium-term prognosis.

## Figures and Tables

**Table 1:** Clinical and immunological parameters of hospitalized COVID-19 patients. All data are reported as mean ± standard deviation.

	Mild to Moderate	Severe COVID-19	*p*-Values
	COVID-19 (n = 94)	(n = 14)	
Gender (M/F) (%)	61/33 (64)	8/6 (57)	0.56
Age (years)	69 ± 14.4	73 ± 11.8	0.87
Clinical symptoms at admission:			
- Fever	68	10	1
- Dyspnoea	50	11	0.08
- Cough	25	4	1
- Phlegma	8	3	0.15
- Gastrointestinal symptoms	34	7	0.38
- Chest pain	5	1	0.57
- Other	40	8	0.39
Chest X rays features:			
- Bilateral pneumonia	58	10	0.04
- Monolateral pneumonia	20	3	
- No pneumonia	16	1	
Comorbidities			0.01
(yes/no)	69/34	6/8	
- Respiratory diseases	15/79	1/13	
- Diabetes	30/64	1/13	
- Cardiomyopathies	35/59	3/11	
- Kidney failure	19/75	0/14	
- Cancer	22/72	1/13	
Blood analysis:			
- Haemoglobin (g/dL)	12.75 ± 1.9	13.12 ± 1.7	0.57
- Haematocrit (%)	40.1 ± 2.2	38.9 ± 1.2	0.98
- White blood cells (10^3^/mm^3^)	4.5 ± 2.2	7.1 ± 1.6	0.32
- Neutrophils (%)	76 ± 12.6	70 ± 8.3	0.12
- Monocytes (%)	5.2 ± 6.5	9.6 ± 7.6	0.04
- Lymphocytes (%)	14 ± 7.3	8.3 ± 4.2	0.005
- Eosinophils (%)	0.3 ± 0.2	0.8 ± 0.4	0.09
- Basophils (%)	0.1 ± 0.1	0.3 ± 0.2	0.08
- Platelets (10^3^/mm^3^)	288 ± 106	198 ± 101	0.16
CRP (mg/dL)	6.3 ± 2.8	12.5 ± 5.8	0.03

## Data Availability

The data presented in this study are available on request from the corresponding author.

## Abbreviations

IL	Interleukin
FVC	Forced vital capacity
FEV1	Forced expiratory volume in the first second
DLCO	Diffusing capacity of the lung for carbon monoxide
